# The Role and Mechanism of Hydrogen-Rich Water in the *Cucumis sativus* Response to Chilling Stress

**DOI:** 10.3390/ijms24076702

**Published:** 2023-04-04

**Authors:** Xue Wang, Zhonghui An, Jiameng Liao, Nana Ran, Yimeng Zhu, Shufeng Ren, Xiangnan Meng, Na Cui, Yang Yu, Haiyan Fan

**Affiliations:** 1 College of Bioscience and Biotechnology, Shenyang Agricultural University, Shenyang 110866, China; 2 Key Laboratory of Protected Horticulture of Ministry of Education, Shenyang Agricultural University, Shenyang 110866, China

**Keywords:** cucumber, hydrogen-rich water, chilling stress, growth and development, photosynthesis, antioxidant system

## Abstract

Cucumber is a warm climate vegetable that is sensitive to chilling reactions. Chilling can occur at any period of cucumber growth and development and seriously affects the yield and quality of cucumber. Hydrogen (H_2_) is a type of antioxidant that plays a critical role in plant development and the response to stress. Hydrogen-rich water (HRW) is the main way to use exogenous hydrogen. This study explored the role and mechanism of HRW in the cucumber defense response to chilling stress. The research results showed that applying 50% saturated HRW to the roots of cucumber seedlings relieved the damage caused by chilling stress. The growth and development indicators, such as plant height, stem diameter, leaf area, dry weight, fresh weight, and root length, increased under the HRW treatment. Photosynthetic efficiency, chlorophyll content, and Fv/Fm also improved and reduced energy dissipation. In addition, after HRW treatment, the REC and MDA content were decreased, and membrane lipid damage was reduced. NBT and DAB staining results showed that the color was lighter, and the area was smaller under HRW treatment. Additionally, the contents of O_2_^−^ and H_2_O_2_ also decreased. Under chilling stress, the application of HRW increased the activity of the antioxidases SOD, CAT, POD, GR, and APX and improved the expression of the *SOD*, *CAT*, *POD*, *GR*, and *APX* antioxidase genes. The GSSG content was reduced, and the GSH content was increased. In addition, the ASA content also increased. Therefore, exogenous HRW is an effective measure for cucumber to respond to chilling stress.

## 1. Introduction

Chilling stress is the main factor that negatively affects plant growth and development and can lead to a series of complex cellular dysfunction and symptoms, including loss of vitality, wilting, chlorosis, infertility, and even death [1,2]. Cucumber is a plant native to the tropics. It likes warmth but does not tolerate cold. The optimum growth temperature is 24 to 28 °C [3]. When the temperature is below 10 °C, cucumber is usually subject to chilling damage [4]. Chilling stress will cause varying degrees of harm to different stages of cucumber. For example, the seeds do not germinate or germination is delayed, the leaves wither in the seedling stage, and necrotic spots appear. It also causes a lower fruiting rate, and the fruit are more like deformed melons. Thence, it is quite important to improve cucumber’s resistance to chilling stress.

At present, cucumber copes with chilling stress mainly through the cultivation of chilling-resistant varieties and the spraying of some exogenous substances (such as nitrogen fertilizer and salt acid leaf surface fertilizer). The cultivation of chilling-resistant varieties takes a long time, while some exogenous substances hurt the environment and the human body. Therefore, finding new types of harmless exogenous products is the focus of this research.

Hydrogen (H_2_) is an odorless, nontoxic dual-atomic gas. Chlamydomonas is the main producer of H_2_ [5,6]. It can be used as a signaling molecule to regulate plant growing development and participate in the plant stress response network [7]. H_2_ was first found to promote seed germination [8]. H_2_ can affect the height of the plant, the elongation of the main root, and the occurrence of side roots. The main supply form of H_2_ is hydrogen-rich water (HRW). HRW has significantly enhanced the germination rate of tomato, cucumber, and winter melon seeds [9]. HRW can improve the fresh weight, hypocotyl, and root length of mung bean seedlings by regulating the level of endogenous gibberellin and auxin [10]. H_2_ can significantly increase the side root growth of cucumber by inducing signaling molecules such as HO-1, CO, and NO [11]. HRW promotes the formation of bulblets in Lilium davidii var. by regulating the metabolism of sucrose and starch [12].

A good deal of research has shown that H_2_ plays a critical role in plant resistance. The earliest research found that H_2_ can participate in the regulation of the HO-1 signal system, significantly improving the oxidation stress of alfalfa caused by paraquat [13]. HO-1 has been proven to be involved in a range of abiotic stress responses in plants, including salt, heavy metals, ultraviolet light, and drought [14,15]. Furthermore, H_2_ plays a significant role in plants in response to osmotic pressure, low temperature, salt stress, and heavy metal stress, through a certain relief effect. The tolerance of rapeseed (*Brassica napus* L.) seedlings to salt, drought, or Cd stress can be enhanced under HRW treatment [16].

H_2_ mainly aids in plant resistance by improving the antioxidant capacity and increasing the stability of the cell membrane, which helps cope with stress. Under low-temperature conditions, spraying HRW on lilies can increase the output of flower buds. H_2_ can reduce active oxygen levels and reduce penetration and lipid peroxidation, thereby improving the cold resistance of bud browning [17]. HRW regulates the decrease of ROS level and alleviates GA-induced programmed cell death in the wheat aleurone layer [18].

There is less research on the role of hydrogen in cucumber resistance, especially in response to chilling stress. Therefore, this study used Jinyan 4 as the test material. First, some morphological indicators, such as plant height, stem diameter, root length, and photosynthesis indicators, were used to research the role of HRW. Then, the mechanisms by which the activity and gene expression of some antioxidases, as well as antioxidants such as ascorbate and glutathione, activated oxygen metabolism were determined. The role and mechanism of HRW in cucumber’s response to chilling stress were identified. This helped to determine whether HRW, a source of H_2_, is a clean fertilizer that can be widely used in agricultural production, which could improve crop yield, enhance plant vitality, reduce the use of pesticide chemical fertilizers, and promote the development of green agriculture.

## 2. Results

### 2.1. HRW Can Enhance Cucumber Growth and Development under Chilling Stress

**Figure 1:**
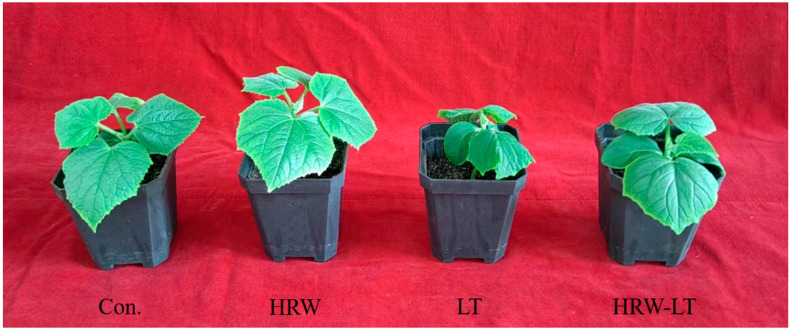
The phenotype of cucumber seedlings under chilling stress after HRW treatment. Con.: control; HRW: treatment by hydrogen-rich water; LT: treatment by chilling stress; HRW-LT: chilling stress after HRW treatment.

**Figure 2:**
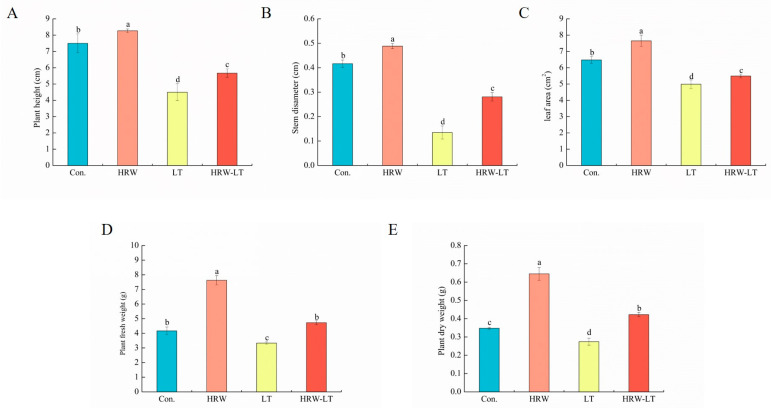
HRW enhanced cucumber growth and development under chilling stress. (**A**) plant height; (**B**) stem diameter; (**C**) leaf area; (**D**) fresh weight; (**E**) dry weight. Values are the means ± SD, *n* = 9 (number of samples). The different letters indicate a significant difference (*p* < 0.05). These indicators were measured after 72 h of chilling stress.

HRW can promote the growth and development of cucumber seedlings. As Figure 1 shows, cucumber seedlings in the HRW treatment group were more robust, compared with the control group. Chilling stress can disrupt the growth and development of cucumber. Cucumber seedlings were shorter than the control group after chilling stress. HRW can mitigate this damage. Compared with those of the control group, the chilling stress treatment significantly decreased plant height, stem diameter, leaf area, fresh weight, and dry weight by 40%, 67.5%, 22.9%, 20.1%, and 21.3%, respectively. HRW+LT treatment dramatically increased these indices by 24.4%, 108.1%, 10%, 41.7%, and 53.4%, significantly, compared to those of the chilling stress-treated plants (Figure 2A–E).

**Figure 3:**
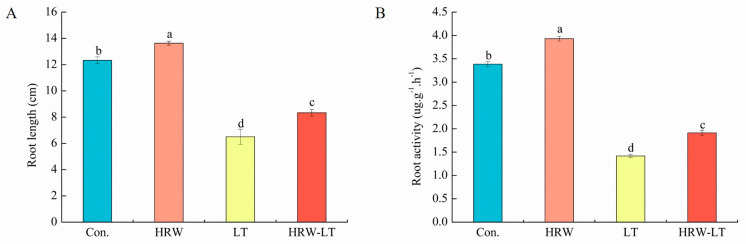
HRW enhanced root system under chilling stress. (**A**) root length; (**B**) root/shoot ratio. Values are the means ± SD, *n* = 9 (number of samples). The different letters indicate a significant difference (*p* < 0.05). These indicators were measured after 72 h of chilling stress.

The root system is the first organ to experience chilling stress. We found that all root indicators, including root length, root/shoot ratio, root fresh weight, root dry weight, root surface area, root volume, and root diameter, were separately lower than those of the control group under chilling stress treatment. They decreased by 47.1%, 64.5%, 62.1%, 54.5%, 43.3%, 18.81%, and 33.6%, respectively. However, after LT+HRW cotreatment, these indices were increased by 27.7%, 70.8%, 147%, 60%, 28%, 12.1%, and 27.7%, respectively, compared with those of the single chilling stress treatment (Figure 3A,B and Figure S1A–E). In addition, the root vitality results show that HRW can help cucumber slow down the damage caused by chilling stress. The root staining of cucumber seedlings was the lightest under chilling stress, which indicated that root vitality was the weakest (Figure S2A). The root vitality increased by 34.8% under LT+HRW cotreatment compared with that under chilling stress (Figure S2B).

### 2.2. Effect of HRW on Photosynthesis of Cucumber Seedlings under Chilling Stress

#### 2.2.1. Effect on Pigment Content

**Figure 4:**
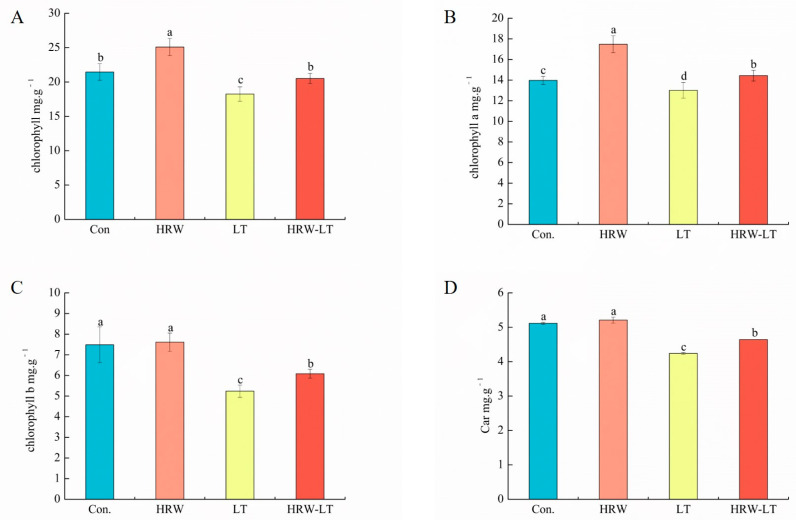
Effect of HRW on pigment content of cucumber seedlings under chilling stress. (**A**) chlorophyll; (**B**) chlorophyll a; (**C**) chlorophyll b.;(**D**) car. Data are the mean ± standard deviation of three biological replicates. The different letters indicate a significant difference (*p* < 0.05). These indicators were measured after 72 h of chilling stress.

Before chilling stress, as compared with the control group, the contents of total chlorophyll and chlorophyll a were increased after HRW treatment. Nevertheless, the contents of chlorophyll b and car were not changed. After chilling stress, the contents of all pigments decreased dramatically. However, the contents of total chlorophyll, chlorophyll a, chlorophyll b, and car were higher than those in the chilling stress group in the LT+HRW cotreatment (Figure 4A–D). This suggests that HRW can slow down the degradation of the chloroplasts of cucumber seedling leaves under chilling stress.

#### 2.2.2. Effect of Gas Exchange Parameters

**Figure 5:**
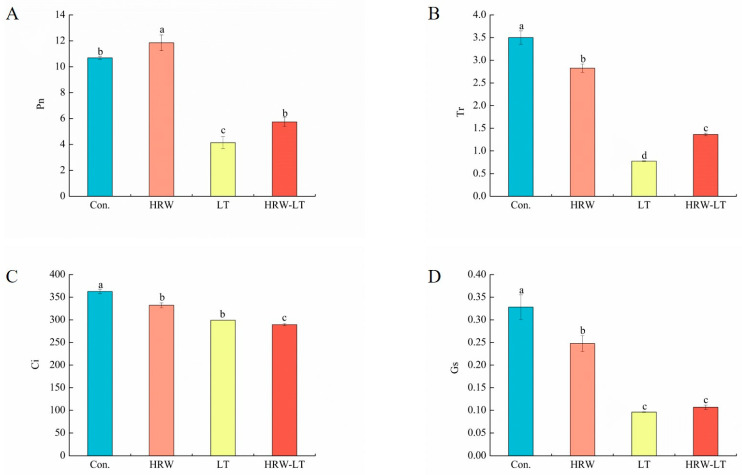
After HRW treatment, the effect of gas exchange parameters of cucumber seedlings under chilling stress. (**A**) Pn; (**B**) Tr; (**C**) Ci; (**D**) Gs. Data are the mean ± standard deviation of three biological replicates. The different letters indicate a significant difference (*p* < 0.05). These indicators were measured after 72 h of chilling stress.

According to the results, we found that the photosynthetic rate (Pn) of cucumber seedlings in the HRW treatment was higher than that in the control group. Compared with that of the distilled water treatment, the transpiration rate (Tr), stomatal conductance (Gs), and carbon dioxide (Ci) of cucumber seedlings in the HRW treatment were lower before chilling stress. After chilling stress, the Pn, Tr, Gs, and Ci were lower. However, the Pn and Tr increased by 38.5% and 76.6%, respectively, after LT+HRW cotreatment compared with the single chilling stress treatment (Figure 5A,B). The Ci was reduced by 3.34%, and Gs was significantly different (Figure 5C,D). These results showed that HRW treatment can cause cucumber seedling leaf cells to maintain high photosynthetic activity, reducing the effect of chilling stress on the photoreactions of cucumber seedlings.

### 2.3. Effect of HRW on the Fluorescence Parameters of Cucumber Seedlings under Chilling Stress

**Figure 6:**
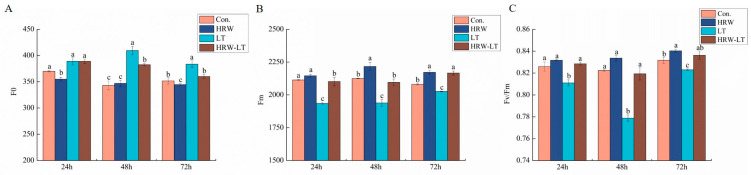
Effect of HRW on the fluorescence parameters of cucumber seedlings under chilling stress. (**A**) F0; (**B**) Fm; (**C**) Fv/Fm. Data are the mean ± standard deviation of three biological replicates. The different letters indicate a significant difference (*p* < 0.05).

Before chilling stress, the initial fluorescence (F0) values at 24 h and 72 h decreased after HRW treatment, and the trend at 48 h was consistent. After chilling stress, the F0 of the three time periods increased. After the HRW+LT treatment, compared with that of chilling stress alone, change was not significant at 24 h. At 48 h and 72 h they were reduced (Figure 6A).

Under normal conditions, Fm did not change at 24 h, but when the processing time increased to 48 and 72 h, the maximum fluorescence (Fm) increased after HRW processing. After chilling stress, both Fm values decreased, but the Fm values at 24 h, 48 h, and 72 h were higher than those of the LT group after HRW+LT treatment (Figure 6B). Before chilling stress, the differences in maximum photochemical efficiency (Fv/Fm) of the two processing methods were not significant at 24 h and 48 h. At 72 h, the Fv/Fm after HRW processing was elevated. Under chilling stress, the Fv/Fm of the three time points decreased, and it showed a trend of falling first and then increasing. Under HRW+LT treatment, the Fv/Fm was increased by 2.1%, 5.3%, and 1.6% at 24 h, 48 h, and 72 h, respectively, compared with the LT stress (Figure 6C).

### 2.4. Effect of HRW on Energy Flow Parameters of Cucumber Seedlings under Chilling Stress

**Figure 7:**
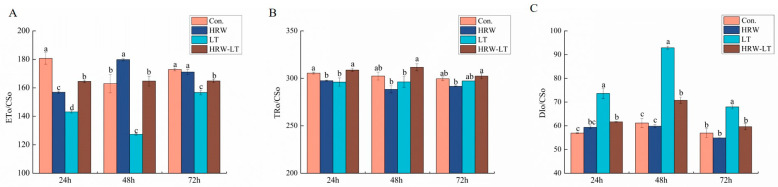
Effect of HRW on energy flow parameters of cucumber seedlings under chilling stress. (**A**) ETo/CSo; (**B**) TRo/CSo; (**C**) DIo/CSo. Data are the mean ± standard deviation of three biological replicates. The different letters indicate a significant difference (*p* < 0.05).

Energy flow parameters include ETo/CSo, TRo/CSo, and DIo/CSo. ETo/CSo is the quantum yield of electron transfer per unit area. TRo/CSo is light energy captured per unit area. DIo/CSo is heat dissipation per unit area. After 50% HRW treatment, the ETo/CSo at 24 h was dramatically reduced compared with that of the control group under normal conditions, but the ETo/CSo at 48 h was increased significantly, while at 72 h it showed no significant change. The changes of TRo/CSo and DIo/CSo at 24 h, 48 h, and 72 h were not obvious. After chilling stress, the ETo/CSo decreased at three time points, but after HRW processing, the ETo/CSo increased significantly (Figure 7A). The TRo/CSo decreased at 24 h and did not change much at 48 h and 72 h. After HRW treatment, the TRo/CSo at 24 h and 48 h was higher and did not change at 72 h (Figure 7B). The DIo/CSo increased obviously and reached the highest value at 48 h under chilling stress (Figure 7C). HRW processing decreased the values at the three time points. These results showed that the energy in the cucumber seedlings after HRW treatment was transformed, which could reduce the chilling stress damage to the cucumber seedlings.

### 2.5. HRW Reduced Cell Membrane Damage in Cucumber Seedlings under Chilling Stress

**Figure 8:**
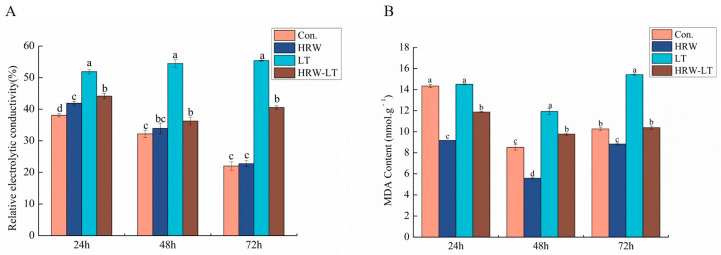
HRW reduced the REC level and MDA content of cucumber seedlings under chilling stress. (**A**) REC; (**B**) MDA content. Data are the mean ± standard deviation of three biological replicates. The different letters indicate a significant difference (*p* < 0.05).

To explore the role of HRW in cell membrane damage under chilling stress, we measured relative electrolytic conductivity (REC) level changes and MDA, hydrogen peroxide (H_2_O_2_), and standard oxygen (O_2_^−^) content changes in cucumber seedlings under chilling stress at 24, 48, and 72 h. As Figure 8A shows, before chilling stress, the REC level of HRW treatment compared with those in the control group increased at 24 h. It did not change at 48 h and 72 h. After chilling stress, the REC level was higher than the control group at three points. It presented an increasing trend with the extension of the chilling stress time. Nevertheless, exogenous HRW treatment of chilling-stressed plants reduced the REC level (Figure 8A).

The MDA content of cucumber seedlings increased gradually after chilling stress, and the trend continued with extended chilling stress time, but the difference was not large. These results indicated that HRW treatment could alleviate this damage at these three time points (Figure 8B).

**Figure 9:**
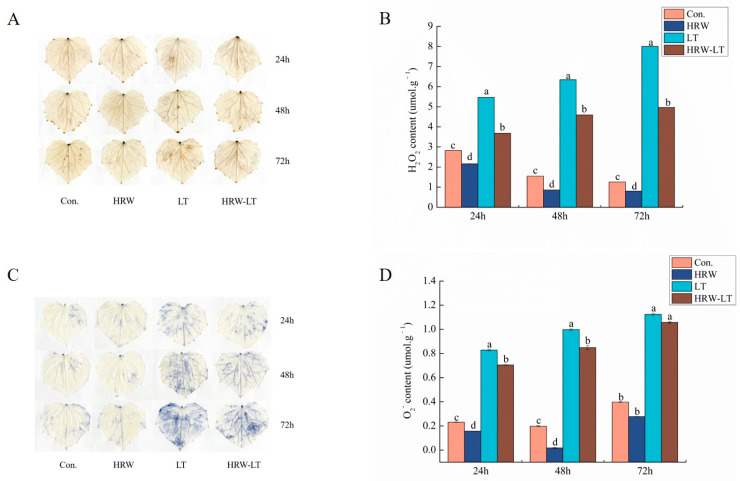
Effect of HRW on the accumulation of H_2_O_2_ and O_2_^−^ of cucumber seedlings under chilling stress. (**A**) DAB staining; (**B**) H_2_O_2_ content; (**C**) NBT staining; (**D**) O_2_^−^ content. Data are the mean ± standard deviation of three biological replicates. The different letters indicate a significant difference (*p* < 0.05).

Diaminobenzidine (DAB) staining showed that the leaves of cucumber seedlings developed brown spots under chilling stress. Over time, the area of brown spots increased (Figure 9A). By comparison, H_2_O_2_ content also increased. Compared with those of the chilling stress group alone, the area of brown spots decreased, and the H_2_O_2_ content was reduced by 32.9–38% with the HRW treatment (Figure 9B). Nitrotetrazolium blue chloride (NBT) staining indicated that blue spots appeared on leaves after chilling stress. The O_2_^−^ content was also enhanced compared with that of the control group. By contrast, the exogenous HRW treatment of chilling-stressed plants reduced spot area and O_2_^−^ content early in the chilling stress (Figure 9C). The content of O_2_^−^ was decreased by 14.9%. The spot area and O_2_^−^ content also decreased at 72 h, but this difference was not significant (Figure 9D).

### 2.6. Effect of HRW on the Antioxidation System of Cucumber Seedlings under Chilling Stress

#### 2.6.1. Effect of HRW on the Enzymatic System of Cucumber Seedlings under Chilling Stress

**Figure 10:**
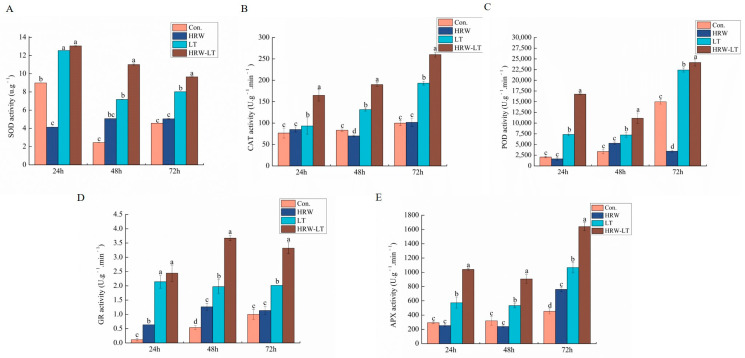
Effect of HRW on the enzymatic system of cucumber seedlings under chilling stress. (**A**) SOD; (**B**) CAT; (**C**) POD; (**D**) GR; (**E**) APX. Data are the mean ± standard deviation of three biological replicates. The different letters indicate a significant difference (*p* < 0.05).

The antioxidant system mainly regulates the plant fat peroxidation process. Figure 10A shows that the SOD enzyme activity after HRW treatment decreased at 24 h before chilling stress, and the difference between 48 h and 72 h was not obvious. After chilling stress, the SOD enzyme activity at 24 h, 48 h, and 72 h was increased. Compared with the chilling stress alone, the enzyme activity at three time points of HRW+LT treatment was increased (Figure 10A). The CAT enzyme activity showed a rise at normal temperature. After HRW treatment, it only decreased at 48 h, and was not changed at other time points. The CAT enzyme activity at 24 h, 48 h, and 72 h increased after chilling stress. After HRW treatment, the enzyme activity was significantly higher (Figure 10B). The activity of the POD enzyme showed no change at other time points, except 72 h at normal temperature. However, under chilling stress, the enzyme activity of the HRW treatment was higher than that of the LT treatment alone (Figure 10C). In addition, the activity was greatly improved after chilling stress.

Figure 10D shows that, before chilling stress, the GR enzyme activity in plants treated with HRW was higher than that in the control at 24 h and 48 h, but there was no significant difference at 72 h. After chilling stress, the enzyme activity at the three time points was increased, and the GR enzyme activity at 48 h and 72 h was greatly improved under the HRW+LT treatment. The improvement at 24 h was not significant (Figure 10D). The activity of APX enzymes was not different, except at 72 h at normal temperature. Under chilling stress treatment for 24 h, 48 h, and 72 h, compared with that of distilled water treatment, the activity of APX treated with HRW significantly improved (Figure 10E).

In summary, HRW treatment is able to improve the activity of SOD, CAT, POD, GR, and APX under chilling stress compared with distilled water treatment, which is conducive to the removal of active oxygen and reduces oxidation damage under chilling stress.

#### 2.6.2. Effect of HRW on the Nonenzymatic System of Cucumber Seedlings under Chilling Stress

**Figure 11:**
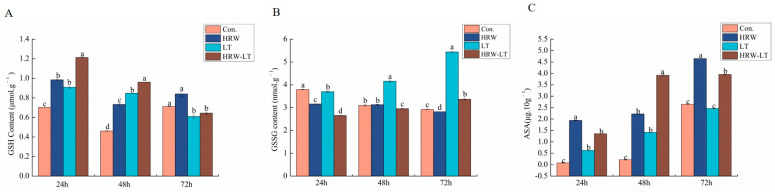
Effect of HRW on the nonenzymatic system of cucumber seedlings under chilling stress (**A**) GSH; (**B**) GSSG; (**C**) ASA. Data are the mean ± standard deviation of three biological replicates. The different letters indicate a significant difference (*p* < 0.05).

At normal temperature, whether in the control group or the processing group, the glutathione (GSH) content showed a trend of first falling and then elevating. The content at 24 h and 48 h was higher than that in the control group, while it did not change at 72 h after HRW treatment. After chilling stress, it showed a trend of decreasing three times, whether in the LT treatment group or the HRW+LT treatment group. After HRW+LT treatment, it was increased by 33.5% and 13.4% compared with separate chilling stress treatment at 24 h and 48 h, respectively, but the content was not much different at 72 h (Figure 11A). Glutathiol (GSSG) content showed a downward trend over time before chilling stress. After chilling stress, the content of GSSG at 24 h, 48 h, and 72 h was increased by 16.9%, 33%, and 92.5%, respectively, compared with that of the control group; HRW processing reduced the content of GSSG by 28.1%, 29.1%, and 38.1%, respectively (Figure 11B). The content of GSH+GSSG and the comparison of GSH/GSSG were improved compared with the separate chilling stress treatment (Figure S3A,B). After chilling stress treatment, the ascorbic acid (ASA) content increased at 24 h and 48 h compared with that of the distilled water treatment. However, it did not change much at 72 h. After the root application of HRW, the ASA content did not change significantly at 24 h, but the contents at 48 h and 72 h increased obviously (Figure 11C).

#### 2.6.3. Effect of HRW on Antioxidant-Related Enzyme Gene Expression in Cucumber Seedlings under Chilling Stress

**Figure 12:**
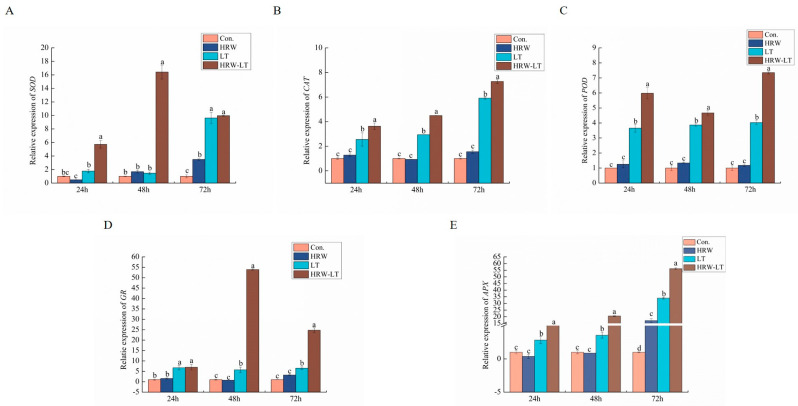
Effect of HRW on antioxidant-related enzyme gene expression in cucumber seedlings under chilling stress. (**A**) *SOD*; (**B**) *CAT*; (**C**) *POD*; (**D**) *GR*; (**E**) *APX*. Data are the mean ± standard deviation of three biological replicates. The different letters indicate a significant difference (*p* < 0.05).

The figure shows that the gene expression of *SOD*, *POD*, *GR*, and *APX* in the control group and processing group did not change significantly before chilling stress, but the expression of *SOD* and *APX* increased at 72 h. After chilling stress, the five genes increased compared with the control group. SOD reached its highest level at 72 h, and there was not much change at 24 h and 48 h (Figure 12A). The expression of *CAT* appeared to cause a trend of elevating three times, which also reached the highest level at 72 h (Figure 12B). The expression of *POD* and *APX* showed a trend of elevation (Figure 12C,E). *GR* expression also increased and reached its highest level at 48 h (Figure 12D). After HRW treatment, the expression of *SOD*, *CAT*, *POD*, *GR*, and *APX* was greatly improved, but the trend between the five genes was not the same.

## 3. Discussion

Chilling stress is a vital factor affecting the quality and yield of cucumber. Plant hormones are signaling compounds that regulate growth, development, and response to environmental stress [19]. Adding exogenous CK to Arabidopsis seedlings can improve their freeze resistance. Under low temperature stress, the CRF gene can promote the germination and formation of lateral roots [20]. BRs can participate in regulating plant growth and development, and help plants adapt to the environment [21]. Exogenous application of BR or a change of its biosynthesis and signal transduction can improve crop yield [22]. Eremina et al. showed that BRs regulated the freezing tolerance [23]. After spraying BRs, the toxicity of heavy metals to rice decreased [24]. H_2_ is a twin-atomic gas that is colorless and tasteless, has a minimum molecular weight, is nontoxic and harmless, has a high heat capacity, and a low density. It has a profound impact on cell activity [25]. The earliest H_2_ was gradually developed into a new type of medical gas. However, recent research has found that H_2_ can participate in regulating the growth metabolism of plants and anti-inverse processes, especially in some abiotic stresses, and may have a key role [26].

Some researchers have shown that H_2_ can promote the growth and development of plants and alleviate the damage caused by abiotic stress. This study found that under normal temperature, the plant height, stem diameter, leaf area, dry weight, and fresh weight of the cucumber seedlings increased compared to those of the control group (Figure 1), and some root indicators also improved, such as root/shoot ratio and root activity (Figure 2). This may be due to the regulation of gene expression, in which HRW is involved in regulating the accumulation of organic matter content as a signaling molecule. After chilling stress, these growth and development indicators were reduced compared with those of the control group. Compared with chilling stress treatment alone, these indicators were significantly increased. These results indicated that HRW treatment relieved the damage to the cucumber seedlings. Likewise, Zeng et al. found that low-concentration H_2_ treatment can promote the growth of rice and mung bean roots and stems, but high-concentration H_2_ treatment had the opposite result, which indicates that H_2_ can participate in the regulation of endogenous hormone levels to regulate rice and mung bean growth and development [27].

Chlorophyll is the central pigment of photosynthesis and has high photoactivity [28]. One of the key factors affecting photosynthesis is changes in the chlorophyll content [29]. Chlorophyll is sensitive to abiotic stress. It is very easy to degrade and may lead to reduced photosynthetic capacity [30]. In the process of photosynthesis, chlorophyll a and chlorophyll b can capture light energy. However, chlorophyll a can convert light energy to electrical energy, so promotion of photosynthesis occurs because chlorophyll a plays a main role. In this study, the contents of chlorophyll, chlorophyll a, and chlorophyll b in cucumber seedlings were reduced. After HRW treatment, although the contents of chlorophyll, chlorophyll a, and chlorophyll b could not be restored to the control amount, they were significantly higher than those under chilling stress alone. Moreover, the content of chlorophyll a in cucumber leaves was higher than that of chlorophyll b, which also confirms that chlorophyll a may be the main pigment that promoted photosynthesis.

Photosynthesis is the basis of plant growth and development and is sensitive to abiotic stress [31]. Chilling will harm the photochemical mechanism of plants, resulting in the reduction of optical capture capabilities, optical chemical efficiency, and optical key enzyme activity, eventually leading to a reduction in the optical rate [32]. When the Pn decreases, if the Gs and Ci decrease at the same time, then the factor limiting Pn is mainly stomata. If Gs and Ci decrease differently, it is not a pore factor [33]. The stomatal closure leads to the reduction of photosynthesis due to the limited diffusion of CO_2_ to the leaves [34,35]. In this study, the Pn decreased, and the Gs and Ci also decreased at the same time after chilling stress, which indicated that chilling stress limited the opening of the pores of the cucumber leaves. After HRW treatment, the degree of reduction of Pn, Gs, and Ci decreased. In addition, the Tr was also reduced compared to that of the control group after chilling stress, but after HRW treatment, the Tr increased compared with that of the chilling stress treatment. The above results showed that HRW could help cucumber leaves maintain high photosynthetic activity. Zhang et al. found that H_2_ treatment reduced the light inhibitory effect of high light exposure on corn leaves and at the same time accelerated the net light rate of the blades, thereby improving the lighting efficiency of PSII [36].

The chlorophyll fluorescence parameter is the point of reflecting the essential section of the plant leaf tablets that can be absorbed and used in light [37]. The results of this study showed that Fm and Fv/Fm decreased, and F0 increased under chilling stress. After HRW treatment, the degree of reduction in Fm and Fv/Fm decreased, and the degree of exaltation in Fm and Fv/Fm decreased. In addition, some energy parameters, such as ETo/Cso and Tro/Cso, also increased, and DIo/CSo decreased under the HRW treatment. This result showed that HRW could adjust the optical chemical activity of cucumber leaves under chilling stress, increase the openness of the PSII reaction center, and maintain a higher electronic transfer rate. The heat dissipation consumption of cucumber seedlings was reduced, and the energy of the cucumber seedlings was absorbed and transmitted after chilling stress.

Reactive oxygen species (ROS) plays an important role in cell signal transduction and body balance [38]. It mainly includes hydrogen peroxide (H_2_O_2_) and superoxide anion (O_2_^−^) [39]. Under the condition of no stress, the generation and clearance of ROS are in a state of dynamic balance in the plant cells, but under chilling stress, the plant reduces the ability to regulate ROS. H_2_O_2_ and O_2_^−^ will accumulate within the cell [40]. When the ROS level is not within the scope of what is involved in the defense mechanism, lipid peroxidation will occur, thereby increasing the permeability of the cell membrane, making electrolytes exit, and accumulating the active oxygen metabolic product MDA. This study found that the content of MDA in cucumber seedlings and the REC increased significantly after chilling stress. H_2_O_2_ and O_2_^−^ contents were also higher than the control group. The DAB and NBT staining of cucumber leaves become darker. After the root application of HRW, these indicators were reduced. DAB and NBT staining also confirmed this result. Previous researchers have found that HRW can reduce the active oxygen content of radish flower buds, thereby enhancing its resistance to stress [41].

A defense system will be established to resist ROS during stress events. There are two types of protection systems in plants. One is the enzymatic system, including SOD, CAT, POD, GR, and APX. Under chilling stress, SOD can convert the active oxygen produced by plants to H_2_O_2_, and then CAT and POD can be removed and cleared [42]. GR and APX are the essential enzymes participating in the ASA-GSH cycle. The GR enzyme can directly inhibit the production of active oxygen. The other is a nonenzymatic system, including ascorbic acid and glutathione [43,44]. ASA and GSH removal of active oxygen will generate DHA and GSSH. GSSH is restored by NADPH to GSH. ROS is cleared cyclically [45]. The results of this study showed that the SOD, CAT, POD, GR, and APX of the enzymatic system improved, the content of ASA and GSH decreased, and the GSSH content increased after chilling stress. Compared with that of the chilling stress alone, the activity of the antioxidant-related enzymes of cucumber seedlings treated with HRW respectively improved. The ASA and GSH contents of the nonenzyme-proliferation systems increased obviously, the GSSH content was reduced, and GSH+GSSH and GSH/GSSG both improved dramatically. This proved that the antioxidant system of cucumber seedlings could be regulated by HRW to promote the clearance of active oxygen under chilling stress. In addition, Ma et al. found that when HRW was applied, the SOD and CAT activities of rice increased, and the PB and CD contents in rice roots were reduced to alleviate the inhibitory effects of CD and PB toxicity [46].

SOD, CAT, POD, APX, GR, and other related antioxidants are controlled by related enzyme genes. H_2_ can improve the expression of antioxidant enzyme genes (such as *OsFeSOD* and *OsCAT*) to help rice resist salt stress [27]. H_2_ can also increase antioxidant genes expression to help *Medicago sativa* resist cadmium stress [47]. These genes include *Cu*, *Zn-SOD*, *POD*, *APX2*, *GPX*, *GS*, *GR*, and so on. This study also found that the enzyme gene expression of cucumber seedlings treated with HRW was significantly increased compared to that under different chilling stress treatment durations. HRW can further regulate the activity of enzymes by regulating the expression of related antioxidant genes to adjust the adaptation of cucumber seedlings to chilling adversity.

## 4. Materials and Methods

### 4.1. Preparation of Hydrogen-Rich Water

One hydrogen rod was soaked in a sealed bottle with 2 L distilled water. After 5 h, hydrogen-rich water was obtained, and the saturation was considered 100%. AN amount of 2 L distilled water was added to the sealed bottle to obtain 50% hydrogen-rich water. The hydrogen rod was provided by Shenyang Yixin Health Service Hydrogen Technology Co. Ltd. (Shenyang, China).

### 4.2. Plant Material

The *Cucumis sativus* variety used was Jinyan 4, which was produced by Tianjin Hongfeng Vegetable Research Co., Ltd. (Tianjin, China). Cucumber seeds were planted in a plastic flowerpot with a diameter of 70 mm and a culture medium (peat soil: vermiculite = 2:1). A plastic flowerpot has one seed. Cucumber plants were grown in a room with a day/night temperature of 25 °C/15 °C and a 16 h light photoperiod. Light intensity was 20,000 Lux. When the cucumber seedlings developed a cotyledon and one leaf and had the same amount of growth, they were separated into two groups. One group received 30 mL 50% HRW to the roots 3 times per day for 5 days. The other group received the same treatment with distilled water. Twenty-four hours after the last water treatment, the cucumber seedlings were put in the light box for cultivation, and the indicators were measured at the three time periods of 24 h, 48 h, and 72 h. The low temperature for cultivation was 15 °C/6 °C (day/night), and the normal temperature was 25 °C/15 °C (day/night).

### 4.3. Growth and Development Indicator Measurements

Four groups of cucumber seedlings (Con., HRW, LT, HRW-LT) of 9 plants each were used to measure growth and development indicators. Plant height and the main root length were measured using a ruler (accurate to millimeters). Stem diameter was measured with a card ruler. The dry weight, fresh weight, root fresh weight, and root dry weight were weighed with an electronic balance (accurate to 0.0001 g), and the root crown ratio was calculated. The leaf area was measured with a weight method. The root scanner was used to determine the root diameter, root area, and root volume of the root system. Root activity was measured by the 1-naphthylamine method [48].

### 4.4. Measurement of Gas Exchange Parameters

The best functional leaf of cucumber seedlings was used to measure Pn, Tr, intercellular concentration of Ci, and Gs with li-6800 (LI-COR, NE, America). The temperature was 25 °C, the humidity was 85%, the carbon dioxide concentration was 350–360 µL·L^−1^, and the light intensity was 800 µmol·m^−2^S^−1^. Six duplications were performed in each group.

### 4.5. Determination of Chlorophyll Content and Chlorophyll Fluorescent Parameters

The chlorophyll content was determined by the ethanol-acetone soaking method, and ethanol, alcohol, acetone, and distilled water extracts were mixed together at a ratio of 4.5:4.5:1. Blades (0.1 g) were cut and placed in 10 mL of extract. The blank was set aside at room temperature until the leaves were white. The solution was analyzed at 663, 645, and 440 nm, and then the formula was used to calculate the content. A portable chlorophyll fluorescence instrument (Hansatech, Taian, China) was used to determine the various fluorescent parameters of the best functional leaf of the cucumber seedlings. After dark treatment for 30 min, the Fm, Fv/Fm, and F0 were measured in the dark. Six duplicates were used for each group.

### 4.6. MDA Content and REC Measurement

MDA was measured using a sulfurbal acid method. Leaf samples (0.5 g) were homogenized in 5 mL 5% trichloroacetic acid (TCA) and ground. The mixture was shaken at 3000 rpm for 10 min until it was clear. After adding 2 mL thiobarbituric acid (TBA) to the 2 mL liquid, the mixture was boiled at 100 °C for 30 min, and the suction value was measured at 450, 532, and 600 nm after cooling. The formula was used for calculation. MDA = [6.45(A532 − A600) − 0.56A450] × N × W^−1^ N: Volume of extract (mL) W: Fresh weight of plant tissue (g).

The REC measurement method was as follows: leaves were washed with tap water and exfoliating water. Then, filter paper was used to dry the samples. A round piece was obtained using a pitch with a diameter of 6 mm. Each test tube received 1 g of leaf material. Ten milliliters of ionic water was added to each tube. They were placed in a vacuum drying box for 10 min and shaken at 180 rpm for 1 h, and the conductive value of L1 was measured. After the measurement, it was placed in boiling water and boiled for 10 min. After cooling, the conductive value L2 was determined, and REC = L1/L2.

### 4.7. H_2_O_2_ and O_2_^−^ Measurement

DAB staining: the leaves were homogenized in DAB staining solution, which contained 50 mg DAB powder, 45 mL distilled water, 25 µL Tween 20 solution, and 2.5 mL 0.2 mol·L^−1^ sodium dihydrogen. The vacuum treatment of the blades was performed for five minutes. The leaves were shaken at 80–100 rpm for 4–5 h. Then, the staining solution was poured, and the bleaching solution was added and boiled in water (replacing the bleaching solution) until the leaves were transparent and photos were taken.

The measurement of H_2_O_2_ content was detected using an H_2_O_2_ content assay kit from Solarbio (Beijing, China). The item number was BC3593, and the specifications were 100 T/96 s. According to the kit, 1 mL reagent was added to the ice bath, centrifuged at 8000 rpm at 4 °C for 10 min, and the liquid was placed on ice to be tested. Then, the solution was added to the liquid according to the instructions to determine the absorbance value at 415 nm. The result was calculated with formulas.

NBT staining: the leaves were homogenized in NBT staining solution that contained 1 mg NBT powder and 10 mL phosphate buffer (39 mL 0.2 mol·L^−1^ NaH_2_PO_4_, 61 mL 0.2 mol·L^−1^ Na_2_HPO_4_). The vacuum treatment of the blades was performed for fifty minutes. The leaves were shaken at 80–100 rpm for 4–5 h. Then, the staining solution was poured, and bleaching solution was added and boiled in water (boiled in water for 30 min, replacing the bleaching solution every 10 min) until the leaves were transparent, and pictures were taken.

The O_2_^−^ content was measured using a Solarbio O_2_^−^ content detection kit. The item number was BC1295, and the specifications were 100 T/96 s. One milliliter of extract was added to 0.1 g of cucumber leaves, fully ground, and analyzed. The homogenates were centrifuged at 4 °C and 12,000 rpm for 20 min to obtain the liquid solution. Then, the solution was added to the liquid according to the instructions to determine the absorbance value at 530 nm. Nitrite standard liquid was diluted to 0.25, 0.125, 0.0625, 0.03125, 0.015625, and 0.0078125 µmol·L^−1^ standard solution to obtain the standard curves, which was applied to the 530 nm measurement to obtain the content of O_2_^−^.

### 4.8. Antioxidant System Measurement

The antioxidant activity measurement method was as follows: the first step was the preparation of crude enzyme solution. Leaves (0.5 g) were homogenized in 5 mL of 0.15 mol·L^−1^ phosphate buffer (pH = 7.5) and were ground on ice. The homogenates were centrifuged at 4 °C and 3000 rpm for 10 min to obtain the liquid crude enzyme solution.

SOD enzyme activity measurement was performed using the NBT reduction method. CAT activity was detected by measuring the consumption of hydrogen peroxide in 3 min at a wavelength of 240 nm. The POD activity was used to increase the oxidation at 470 nm for detection.

APX and GR activities measurement methods were as follows: leaves (0.2 g) were added to 1.6 mL of precooled 0.05 mol·L^−1^ phosphate buffer (pH = 7.8) to grind. The homogenates were centrifuged at 4 °C and 12,000 rpm for 20 min to obtain the liquid solution. APX reacts with EDTA-Na_2_, ASA, and H_2_O_2_, and the enzyme activity was determined through the absorbance value at 290 nm in 1 min. GR reacts with NADPH and HEPES, and the enzyme activity was determined through the absorbance value at 340 nm within 1 min.

Measurement of the content of GSSG: The Suzhou Grais Sisi Biotechnology Co., Ltd (Suzhou, China). Kit was used to determine the content of GSSG. The item number is G0207W. Leaves (0.1 g) were homogenized in 1 mL extract solution and ground on ice. The homogenates were centrifuged at 4 °C and 3000 rpm for 10 min to obtain the liquid solution, which was placed on ice to be tested. Then, the standard curve equation in accordance with the kit method was prepared, and the absorbance value of the extract solution at 412 nm was measured. The GSSG content was calculated on the basis of the standard curve equation. The GSH content was also detected using Suzhou Grais Sisi Biotechnology Co., Ltd. The method was the same as GSSG.

ASA measurement was the redox method. Condoric acid can restore Fe^3+^ to Fe^2+^. The rack ion and the red FeCl can react to form a red color. There is an absorption peak at 534 nm. One gram of leaves was added to 4 mL of 50 g·L^−1^ TCA to grind. The homogenates were filtered to obtain the liquid solution. The liquid reacted with the reaction solution, and the light absorption value was measured at 534 nm. The content was determined on the basis of the standard curve equation.

### 4.9. Primer Design and Statistical Analysis

Primer 5 software was used for primer design (Table S1). Statistical significance was analyzed using one-way analysis of variance (ANOVA) (*p* < 0.05) via SPSS software (SPSS 22.0, Nlinedown, Guangzhou, China). Significant differences between means used different letters to indicate.

## 5. Conclusions

The 50% HRW treatment increased growth and development indicators such as plant height, stem diameter, leaf area, fresh weight, dry weight, and root length to slow down the damage caused to cucumber seedlings by chilling stress. By increasing photosynthetic parameters such as chlorophyll content and Pn, the optical chemical activity of cucumber leaves under chilling stress was adjusted. The HRW treatment also reduced the MDA, H_2_O_2_, and O_2_^−^ contents and REC; improved the SOD, CAT, POD, GR, and APX antioxidase activities and gene expression; reduced the ASA and GSH contents; and decreased the GSSH content to reduce the damage caused by active oxygen after chilling stress. This study can help to improve the survivability of cucumber seedlings under chilling stress, thereby improving the quality and yield of cucumber fruits in the future.

## Data Availability

All data presented in article and supplementary materials.

## Supplementary Materials

The following supporting information can be downloaded at: https://www.mdpi.com/article/10.3390/ijms24076702/s1.
