# Supplementary material for: The Role and Mechanism of Hydrogen-Rich Water in the Cucumis sativus Response to Chilling Stress
Source: Int J Mol Sci. 2023 Apr 4;24(7):6702. doi: 10.3390/ijms24076702 (PMC10095547; doi:10.3390/ijms24076702)
Supplement: Supplementary file 1 [file ijms-24-06702-s001.zip › ijms-2194123-supplementary.pdf]

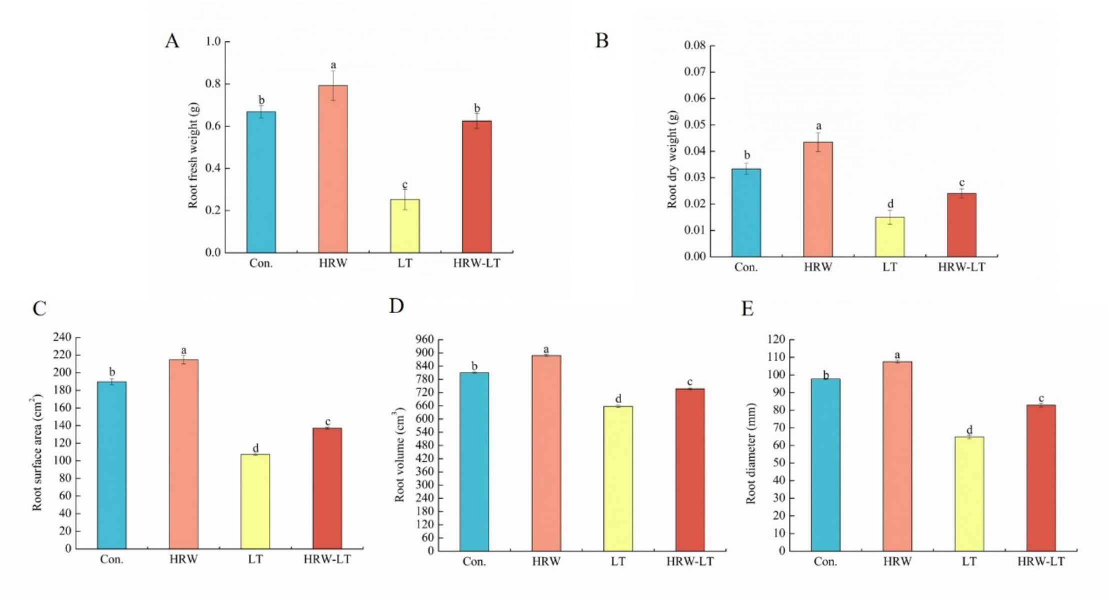

Figure S1. HRW increased cucumber root growth under chilling stress. (A) root fresh weight. (B) root dry weight. (C) root surface area. (D) root volume. (E) root diameter. Values are the means ± SD, n = 9 (number of samples). The different letters indicate a significant difference ($p < 0.05$). These indicators were measured after 72 h of chilling stress.

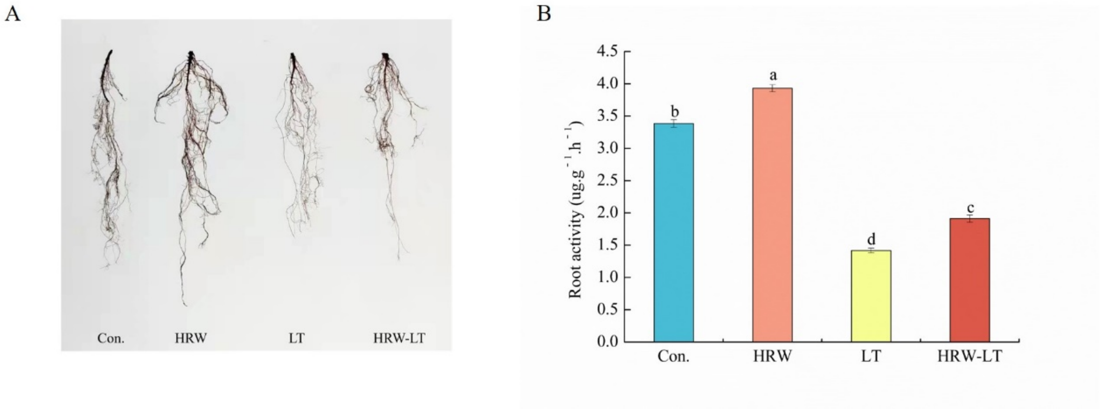

Figure S2. HRW enhanced cucumber root vitality under chilling stress. (A) root staining. (B) root vitality. Data were the mean ± standard deviation of three biological replicates. The different letters indicate a significant difference ($p < 0.05$). These indicators were measured after 72 h of chilling stress.

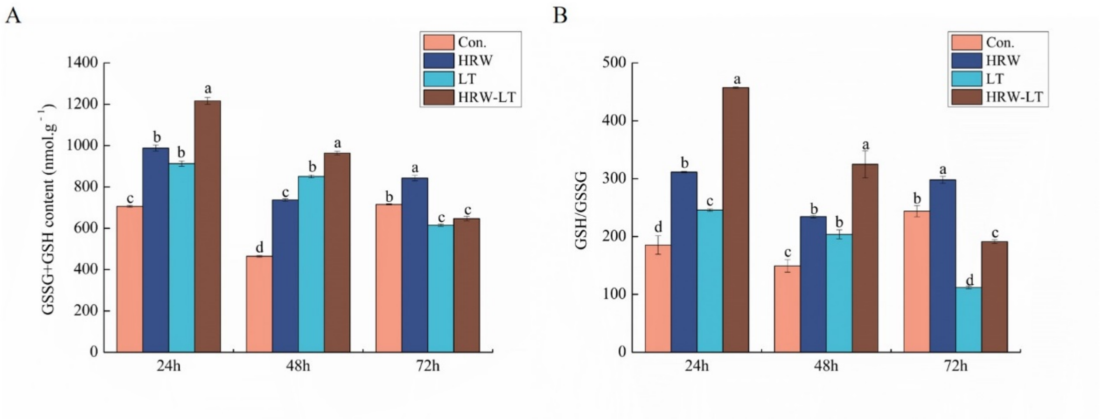

Figure S3. HRW effected cucumber GSH+GSSG content and GSH/GSSG under chilling stress. (A) GSH+GSSG content. (B) GSH/GSSG. Data were the mean ± standard deviation of three biological replicates. The different letters indicate a significant difference ($p < 0.05$).

Table S1 The primers of qRT-PCR used in this study

**Analysis**	**Primer name**	**Sequence (5'-3')**
qRT-PCR for Actin	Actin-F	TCGTGCTGGATTCTGGTG
	Actin-R	GGCAGTGGTGGTGAACAT
qRT-PCR for *SOD*	*SOD*-F	CACAACCAATGGCTGCATGTCG
	SOD-R	ATGGTGAAGTTAGCCTTGCCATC
qRT-PCR for *CAT*	*CAT*-F	ACTTTAAGGAGCCCGGAGAGAG
	CAT-R	CGGATAAATCGTTCCTGCCTGTC
qRT-PCR for POD	*POD*-F	ATCTTGTTGCTCTTTCAGGTAGCC
	POD-R	AGACGTTGCCTGAAGCTAGTGC
qRT-PCR for GR	*GR*-F	GGCGCAGGCCTAATACAAAGAAC
	GR-R	TCTCCAACAGCCCAAATTGAAGG
qRT-PCR for APX	*APX*-F	GGCTATTGGAGCCGATCAAGGAAC
	APX-R	CAGCAACAACACCAGCCAACTG

Table S2 Full names of some nouns

**abbreviation**	**full name**
Pn	photosynthetic rate
Tr	transpiration rate
Gs	stomatal conductance
Ci	carbon dioxide
F0	initial fluorescence
Fm	maximum fluorescence
Fv/Fm	maximum photochemical efficiency
REC	relative electrolytic conductivity
MDA	malondialdehyde
H_2O_2	hydrogen peroxide
O_2^-	standard oxygen
DAB	diaminobenzidine
NBT	nitrotetrazolium blue chloride
SOD	superoxide dismutase
CAT	catalase
POD	peroxidase
APX	ascorbate peroxidase
GR	glutathione reductase
GSH	glutathione
GSSG	glutathiol
ASA	ascorbic acid
